# Unveiling trends and clinical progress of immunotherapy for endometrial cancer: a scientometric and clinical trial landscape analysis

**DOI:** 10.3389/fimmu.2026.1668903

**Published:** 2026-02-09

**Authors:** Ruoyan Liu, Wenhui Shan, Zhuopeng Hu, Hong Wang, Zhening Wang, Lei Yang, Rui Guo

**Affiliations:** 1 Department of Clinical Laboratory, The First Hospital of Jilin university, Changchun, China; 2 The First Hospital of Jilin University, Changchun, China; 3 Cancer Center, The First Hospital of Jilin university, Changchun, China

**Keywords:** endometrial cancer, scientometric analysis, clinical trials, immunotherapy, immune checkpoint blockade

## Abstract

**Background:**

Endometrial cancer (EC) is a heterogeneous and increasingly prevalent malignancy characterized by distinct molecular subgroups that exhibit fundamentally different immune profiles. These immunologic differences shape tumor–immune interactions, influence responsiveness to immunotherapy, and underscore the importance of biologically informed treatment strategies. As the clinical application of immune checkpoint inhibitors expands, understanding the mechanistic and translational landscape of immunotherapy in EC has become essential for guiding precision oncology.

**Methods:**

We systematically retrieved 836 immunotherapy-related publications on EC from the Web of Science Core Collection (1999–2024) and conducted a scientometric analysis using VOSviewer and CiteSpace. Analyses included publication trends, country and institutional collaborations, author networks, and keyword clustering. Furthermore, we screened 391 clinical trials from ClinicalTrials.gov and ICTRP databases to assess the clinical research landscape.

**Results:**

Publication output and clinical trials on EC immunotherapy have shown a continuous upward trend over the past two decades. The United States and China emerged as leading contributors in both publications and pivotal clinical trials. Among the most frequently co-cited references, clinical studies account for a significant proportion, particularly those published in the last five years. The landscape reflects a shift toward immune checkpoint blockade and combination therapy strategies, with some clinical trials demonstrating promising efficacy.

**Conclusion:**

Our integrated scientometric and clinical trial analysis reveals a rapid evolution in EC immunotherapy research, highlighting checkpoint blockade as a central therapeutic approach. The trend toward combination regimens underscores the translational potential of immunotherapy in EC and points toward emerging directions for future research and clinical application.

## 1 Introduction

Uterine corpus cancer is the sixth most frequently diagnosed malignancy among women worldwide, with approximately 417,000 new cases and 97,000 deaths reported in 2020 (1). Incidence is highest in Northern America, Europe, Micronesia/Polynesia, and Australia/New Zealand, whereas mortality disproportionately affects Eastern Europe, Micronesia/Polynesia, the Caribbean, and Northern America. Socioeconomic and racial disparities have been consistently associated with outcome differences in endometrial cancer, with multiple population-based analyses reporting higher mortality among Black women compared with White women (2, 3). Globally, the increasing incidence of EC is largely driven by rising obesity rates and population aging. EC is now recognized as a biologically heterogeneous disease. According to The Cancer Genome Atlas (TCGA), four molecular subtypes—POLE-ultramutated, microsatellite instability–high (MSI-H), copy-number high (CNH), and copy-number low (CNL)—display distinct genomic, immunologic, and clinical features (4). These molecular differences have important therapeutic implications and provide the foundation for precision medicine approaches.

For recurrent or metastatic EC, platinum-based chemotherapy remains the standard first-line regimen, and HER2-positive serous carcinomas may benefit from the addition of trastuzumab. Endocrine therapy—including progestins, aromatase inhibitors, and tamoxifen—can be used in low-grade, hormonally responsive tumors, particularly in older patients or those unable to tolerate chemotherapy. The emergence of immune checkpoint blockade (ICB) has reshaped the systemic treatment landscape, especially for MSI-H or mismatch-repair–deficient (dMMR) tumors, which exhibit high tumor mutational burden, increased neoantigen load, and an inflamed tumor microenvironment. Multiple clinical studies have consistently demonstrated that MSI-H/dMMR tumors respond substantially better to PD-1 pathway inhibition than microsatellite-stable (MSS) or mismatch-repair–proficient (pMMR) tumors (5). These observations have led to the regulatory approval of several PD-1 inhibitors and have stimulated extensive investigation of combination strategies aiming to expand the benefits of immunotherapy beyond the MSI-H/dMMR population.

Despite these advances, the majority of EC cases remain pMMR/MSS and show limited response to ICB monotherapy, highlighting the need for new biomarkers and rational therapeutic combinations. As research momentum increases, integrating epidemiologic trends, molecular classification, and immunologic profiling will be essential for developing more effective and personalized treatment strategies.

## 2 Materials and methods

The Web of Science Core Collection (WoSCC) is the most essential data source for Citespace (6.1.R3) and VOS viewer (1.6.18). The data for this scientometric analysis was obtained from the Science Citation Index Expanded in WoSCC the Science Citation Index Expanded. The search terms were: TS= (Endometrial Cancer OR Endometrial Neoplasms OR Endometrial Carcinoma OR Endometrial Adenocarcinoma) AND TS=(immunotherapy). Document type: articles and reviews. Language: English. Publication years range from 1999 to 2024. The search process was conducted on 7/12/2024. In the end, information from 836 documents was downloaded as plain text files and flat delimited files, respectively. The Web of Science (WoS) platform provides a distribution of publications and citations by year. Citespace (6.4.R1): a total of 836 documents were counted without duplicate values, according to the statistics of the fields showed that 834 with Abstracts (99.760765%), 836 with Digital Object Identifier (100%), 836 with Subject Categories (100%), 710 with Author Keywords (84.9282%).

VOS viewer (1.6.20): 836 results were downloaded from WoSCC as flat delimited files.VOS viewer was used to analyze the authors of studies in this field.

In addition to the scientometric dataset, we also systematically collected immunotherapy-related clinical trials in endometrial cancer from ClinicalTrials.gov and the WHO ICTRP database. Trials were screened to extract study phase, design type, enrollment size, intervention category, target pathway, country, and recruitment status. Interventions were categorized into immune checkpoint inhibitors, targeted therapies, vaccines, adoptive cell therapies, and combination regimens.

To enable quantitative evaluation, annual publication trends, citation counts, and clinical trial registrations were analyzed using linear regression. Differences in the distribution of therapeutic modalities across trials were examined using the chi-square test. Pearson and Spearman correlation analyses were used to assess the association between country-level publication output and clinical trial activity. For therapeutic efficacy comparisons, a logit-transformed random-effects model was applied to pool objective response rate (ORR) in dMMR/MSI-H versus pMMR/microsatellite stable (MSS) subgroups.

The overall design of this scientometric and clinical trial landscape analysis follows an integrative and interdisciplinary research perspective, emphasizing the contextualization of quantitative publication trends within broader mechanistic and translational frameworks. This approach is consistent with recent biomedical research paradigms that aim to bridge data-driven analyses with biological interpretation and clinical relevance, particularly in rapidly evolving oncologic fields (6).

## 3 Clinical trials

In this study, immunotherapy-related clinical trials in endometrial cancer were systematically retrieved from ClinicalTrials.gov and the WHO International Clinical Trials Registry Platform (ICTRP) using the terms “endometrial cancer” and “immunotherapy”. Both registries were searched without restrictions on study phase or recruitment status (7).

Trials were included if they involved patients with endometrial cancer, and evaluated at least one immunotherapy-related intervention. Trials were excluded if they were non-interventional, lacked available intervention information, or were duplicate entries across registries. For duplicated records, the ClinicalTrials.gov entry was preferentially retained.

For each eligible trial, we extracted the NCT number, study phase, design type, enrollment size, country, recruitment status, intervention type, immunotherapy target, and combination regimen. Interventions were categorized into immune checkpoint inhibitors (PD-1/PD-L1/CTLA-4), targeted therapies, vaccines, adoptive cell therapies, and multimodal combinations. Missing or ambiguous intervention descriptions were manually verified against the official registry entry.

To enable analytical comparison across trials, categorical variables (e.g., trial phase, intervention type) were coded into structured fields, and enrollment and country data were standardized. χ² tests were used to compare the distribution of intervention types across study phases. Linear regression was applied to evaluate temporal trends in annual trial registration. Pearson and Spearman correlations were used to assess associations between country-level publication output and clinical trial activity.

## 4 Result

### 4.1 Scientometric analysis

#### 4.1.1 Annual distribution of publications and citations

**Figure 1:**
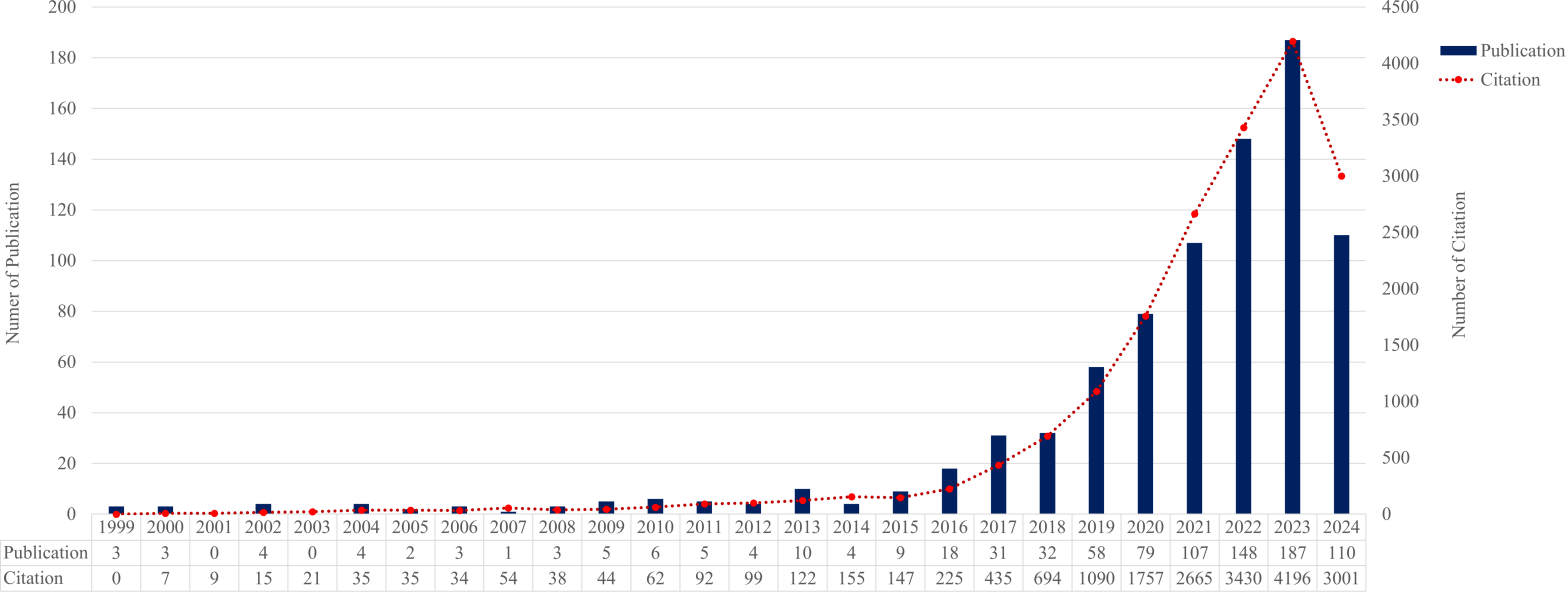
Annual publication and citation trends in immunotherapy for endometrial cancer (1999–2024). The number of publications and total citations related to immunotherapy for endometrial cancer shows a steadily increasing trend over the past two decades. Data were extracted from the Web of Science Core Collection (WoSCC).

Based on 836 publications retrieved from 1999 to 2024, both annual publication output and citation frequency showed a significant upward trajectory. Linear regression analysis indicated that annual publications increased steadily over time (β = 5.14, p = 5.2 × 10^-6^), while annual citation counts also rose markedly (β = 121.94, p = 7.1 × 10^-6^), reflecting growing scientific attention and expanding academic impact in the field of endometrial cancer immunotherapy. These results collectively suggest that the interest and scientific contribution in this domain have accelerated markedly over the past two decades (**Figure 1**).

#### 4.1.2 Relevant countries and institutions in this field

A total of 19 countries contributed to the 836 publications identified in this study. The United States showed the highest level of research productivity, contributing 258 articles, followed by Italy (n = 79), France (n = 41), and Japan (n = 37). This disparity likely reflects differences in research funding, the concentration of comprehensive cancer centers, and the earlier adoption of immunotherapy-related programs in high-income countries.

To examine whether research productivity corresponds to clinical research activity, we further assessed the correlation between publication volume and the number of registered clinical trials across the five most research-active countries (United States, China, Italy, France, and Japan). Spearman’s rank correlation showed no significant association between the two variables (ρ ≈ 0.00, p = 1.0), and Pearson’s correlation similarly demonstrated no meaningful linear relationship (r ≈ 0.20, p = 0.74). These findings indicate that national research output and clinical trial activity are not directly aligned, suggesting differing patterns of scientific emphasis, regulatory environments, and clinical research capacity among countries.

At the institutional level, six of the top ten most productive institutions were located in the United States, highlighting its central role in advancing immunotherapy research in endometrial cancer. Institutions with the highest betweenness centrality in the collaboration network were predominantly major academic medical centers, indicating their structural influence in facilitating international research cooperation. These findings suggest that global research output is not only geographically concentrated but also shaped by institutional capacity and collaborative connectivity.

#### 4.1.3 Author

We analyzed publication output, citation counts, and co-citation relationships to identify influential contributors in this field. Eight authors published more than ten articles, with Lorusso D (19 publications), Zhang Y (17 publications), and Pignata S (15 publications) being the most productive. Citation analysis showed that Pignata S had the highest total citation count (501 citations), followed by Santin AD (328 citations), reflecting their sustained academic impact.

**Figure 2:**
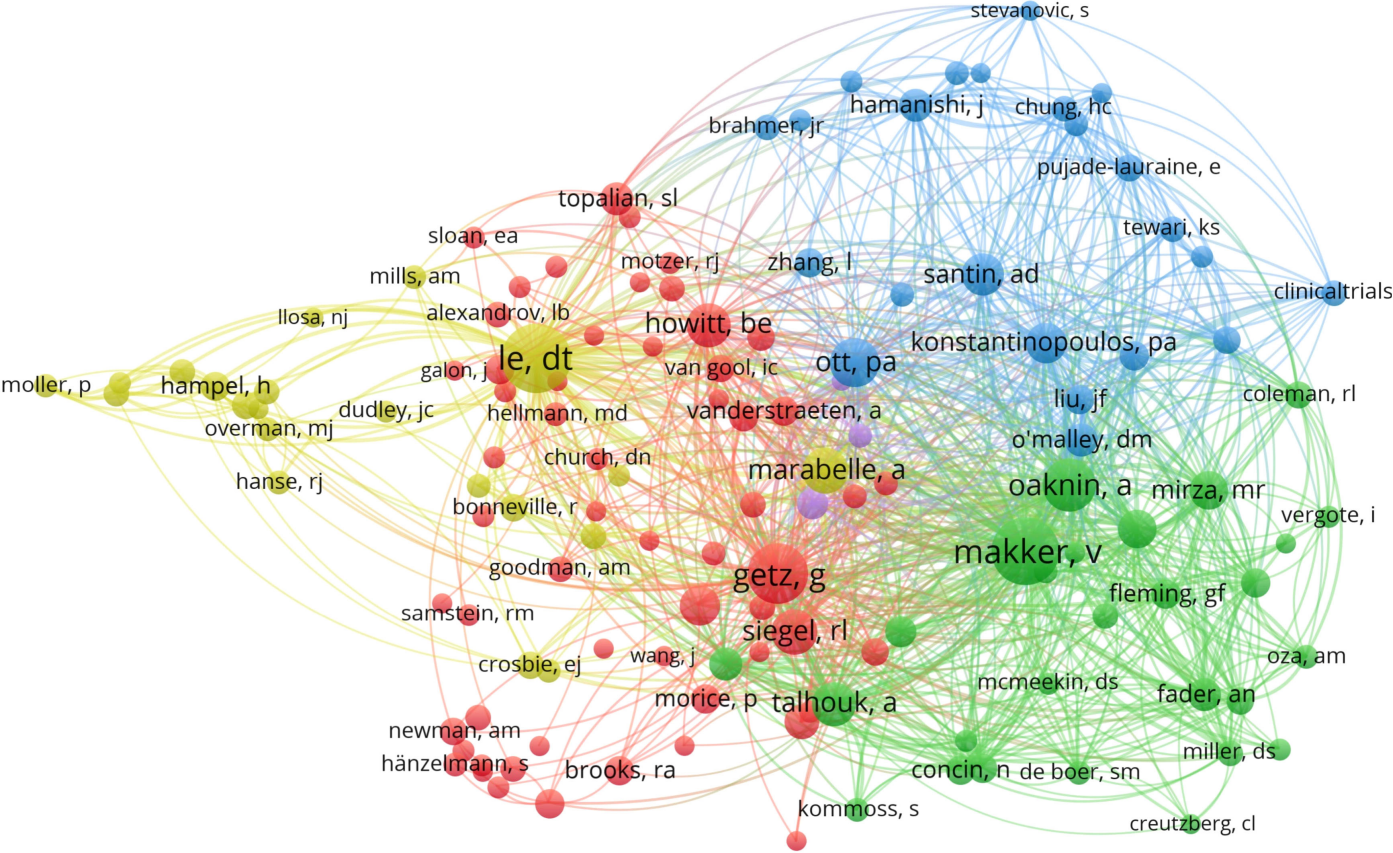
Co-citation network of authors in immunotherapy research on endometrial cancer. The author co-citation analysis was visualized using VOSviewer. Each node represents an author, with node size proportional to the number of times the author was co-cited. The connections between nodes indicate co-citation relationships, and the clustering colors reflect groups of authors frequently cited together. The analysis identified clusters representing closely collaborating or thematically related authors.

Co-citation analysis, which provides a deeper indication of an author’s influence within a specific research domain (8), revealed several distinct collaborative clusters. Using VOSviewer, 132 authors with more than 30 cumulative citations were mapped to construct the co-citation network. As shown in **Figure 2**, authors such as Getz G, Siegel RL, and Yoshihara K formed a closely linked cluster, while Makker V, Oaknin A, and Fader AN formed another major group. Hampel H and Moller P also demonstrated strong co-citation linkages. Fourteen authors were co-cited more than 100 times, with Le DT (325 co-citations), Makker V (325 co-citations), and Getz G (261 co-citations) ranking highest. These co-citation patterns highlight the central role of these investigators in shaping the development of immunotherapy research in endometrial cancer.

#### References

4.1.4

**Figure 3:**
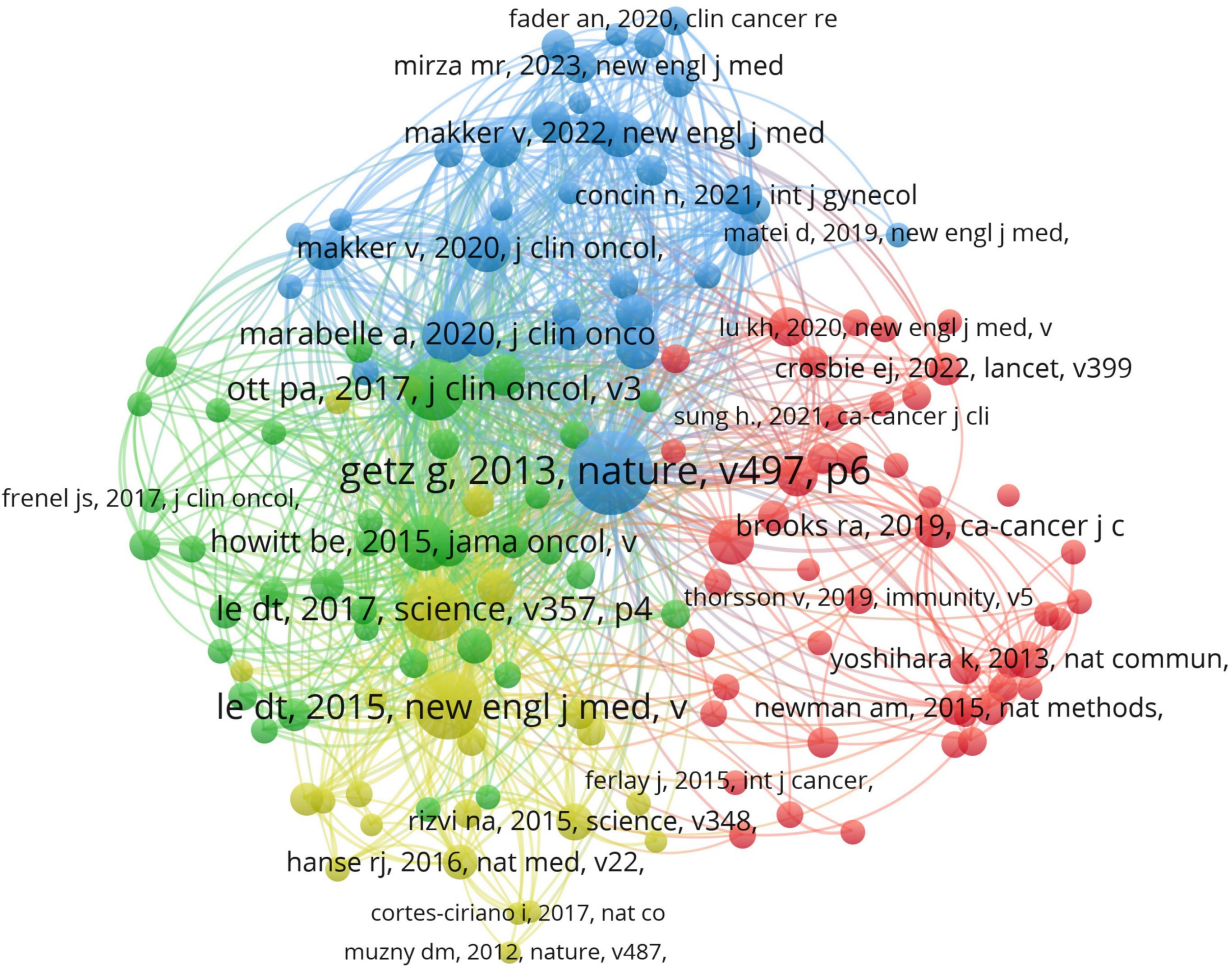
Co-citation network of references in immunotherapy research on endometrial cancer. The network map was generated by VOSviewer based on reference co-citation analysis. Each node represents a cited reference, with node size proportional to its total citation frequency. Lines between nodes indicate co-citation relationships. The clustering colors group references with similar citation patterns. Among the 15 most frequently cited references, 10 were published within the last five years, highlighting recent advances in immunotherapy strategies. Notably, 9 of these key references are clinical trials, including pivotal studies on pembrolizumab, dostarlimab (a bispecific antibody), and the combination of lenvatinib with pembrolizumab.

Co-citation analysis identified the key foundational literature that has shaped the development of immunotherapy for endometrial cancer. Among the 15 most frequently co-cited publications, 10 were published within the past five years, indicating a rapid acceleration of research activity and conceptual consolidation in this field. Notably, nine of these publications were clinical trials, six of which evaluated pembrolizumab-based therapies, reflecting its central role in immunotherapeutic strategies. Other frequently co-cited studies included a Phase I trial of dostarlimab and a Phase II evaluation of lenvatinib combined with pembrolizumab (**Figure 3**, **Table 1**).

**Table 1:** The most cited 15 references.

Year	Title	Type	First author	Journal	Focus and main idea	IF(2024)	JCR	Cocitation
2017	Safety and Efficacy of Pembrolizumab in Advanced,Programmed Death Ligand 1–Positive Cervical Cancer : Results From the Phase Ib KEYNOTE-028 Trial	Clinical Trial	Frenel JS	J Clin Oncol	Pembrolizumab shows promising efficacy and manageable safety in PD-L1–positive advanced cervical cancer.	28.245	Q1	138
2015	Association of Polymerase e-Mutated and Microsatellite-Instable Endometrial Cancers With Neoantigen Load, Number of Tumor-Infiltrating Lymphocytes, and Expression of PD-1 and PD-L1	Research Support	Brooke E Howitt	JAMA Oncol	POLE-mutated and MSI endometrial cancers show high neoantigen loads and increased tumor-infiltrating lymphocytes.	22.4416	Q1	118
2020	Efficacy of Pembrolizumab in Patients With Noncolorectal High Microsatellite Instability/Mismatch Repair-Deficient Cancer: Results From the Phase II KEYNOTE-158 Study	Clinical Trial	Marabelle A	J Clin Oncol	Pembrolizumab is effective for treating advanced MSI-H/dMMR noncolorectal cancers.	28.245	Q1	105
2020	Lenvatinib Plus Pembrolizumab in Patients With Advanced Endometrial Cancer	Clinical Trial	Makker V	J Clin Oncol	Lenvatinib combined with pembrolizumab shows promising results for advanced endometrial cancer, improving survival outcomes.	28.245	Q1	85
2022	Lenvatinib plus Pembrolizumab for Advanced Endometrial Cancer	Clinical Trial	Makker V	N Engl J Med	Lenvatinib combined with pembrolizumab significantly improves progression-free and overall survival in advanced endometrial cancer compared to chemotherapy.	70.67	Q1	75
2011	Global cancer statistics	Article	Jemal A	CA Cancer J Clin	The global cancer burden is rising due to aging populations and increasing adoption of cancer-causing behaviors.	223.679	Q1	72
2016	Endometrial cancer	Review	Morice P	Lancet	Endometrial cancer treatment includes surgery, radiotherapy, chemotherapy, with emerging targeted and immunotherapy options.	59.102	Q1	65
2019	Current recommendations and recent progress in endometrial cancer	Review	Brooks RA	CA Cancer J Clin	Endometrial cancer is rising with evolving treatments including surgery, targeted therapies, and immunotherapy for certain cases.	223.679	Q1	64
1983	Two pathogenetic types of endometrial carcinoma	Article	Bokhman JV	Gynecol Oncol	Two pathogenetic types of endometrial carcinoma are identified based on the presence or absence of endocrine-metabolic disturbances.	4.393	Q2	64
2019	Phase II Study of Avelumab in Patients With Mismatch Repair Deficient and Mismatch Repair Proficient Recurrent/Persistent Endometrial Cancer	Clinical Trial	Konstantinopoulos PA	J Clin Oncol	Avelumab shows promising activity in mismatch repair-deficient endometrial cancer but limited efficacy in mismatch repair-proficient cases	28.245	Q1	62
2020	Clinical Activity and Safety of the Anti-Programmed Death 1 Monoclonal Antibody Dostarlimab for Patients With Recurrent or Advanced Mismatch Repair-Deficient Endometrial Cancer: A Nonrandomized Phase 1 Clinical Trial	Clinical Trial	Oaknin A	JAMA Oncol	Dostarlimab demonstrates durable antitumor activity and an acceptable safety profile in mismatch repair-deficient endometrial cancer.	22.416	Q1	61
2019	Lenvatinib plus pembrolizumab in patients with advanced endometrial cancer: an interim analysis of a multicentre, open-label, single-arm, phase 2 trial	Clinical Trial	Makker V	Lancet Oncol	Lenvatinib combined with pembrolizumab shows promising antitumor activity in advanced endometrial cancer patients.	35.386	Q1	60
2022	Safety and antitumor activity of dostarlimab in patients with advanced or recurrent DNA mismatch repair deficient/microsatellite instability-high (dMMR/MSI-H) or proficient/stable (MMRp/MSS) endometrial cancer: interim results from GARNET-a phase I, single-arm study	Clinical Trial	Oaknin A	J Immunotherapy Cancer	Dostarlimab shows effective antitumor activity with manageable safety in advanced endometrial cancer.	8.676	Q1	57
2022	Pembrolizumab in Patients With Microsatellite Instability-High Advanced Endometrial Cancer: Results From the KEYNOTE-158 Study	Clinical Trial	O’Malley DM	J Clin Oncol	Pembrolizumab showed strong and lasting anti-tumor effects with manageable side effects in patients with advanced microsatellite instability-high (MSI-H) endometrial cancer.	28.245	Q1	57
2022	Global Cancer Statistics 2020: GLOBOCAN Estimates of Incidence and Mortality Worldwide for 36 Cancers in 185 Countries	Article	Sung H	CA Cancer J Clin	Global cancer cases and deaths are increasing, with breast cancer as the most diagnosed and lung cancer as the top cause of death.	223.679	Q1	57

Mechanistic studies were also highly represented among the top co-cited references. A seminal analysis demonstrated that POLE mutations and microsatellite instability are strongly associated with increased neoantigen load, higher tumor-infiltrating lymphocyte (TIL) density, and elevated PD-1/PD-L1 expression, thereby providing a biological rationale for immunotherapy responsiveness. Subsequent clinical evidence strengthened this framework: a Phase II trial in 2020 confirmed the favorable efficacy of pembrolizumab in MSI-H/dMMR endometrial cancer (9), and another Phase II study demonstrated clinically meaningful benefits from lenvatinib plus pembrolizumab in advanced disease, particularly in MSI-H/dMMR subgroups (10).

Conversely, two trials reported substantially lower response rates to dostarlimab and avelumab among pMMR/MSS patients compared with dMMR/MSI-H cohorts, underscoring the selective benefit of immune checkpoint inhibitors in molecularly defined populations. These patterns collectively highlight the close integration of molecular pathology and clinical decision-making in contemporary immunotherapy research for endometrial cancer.

#### 4.1.5 Key words

**Figure 4:**
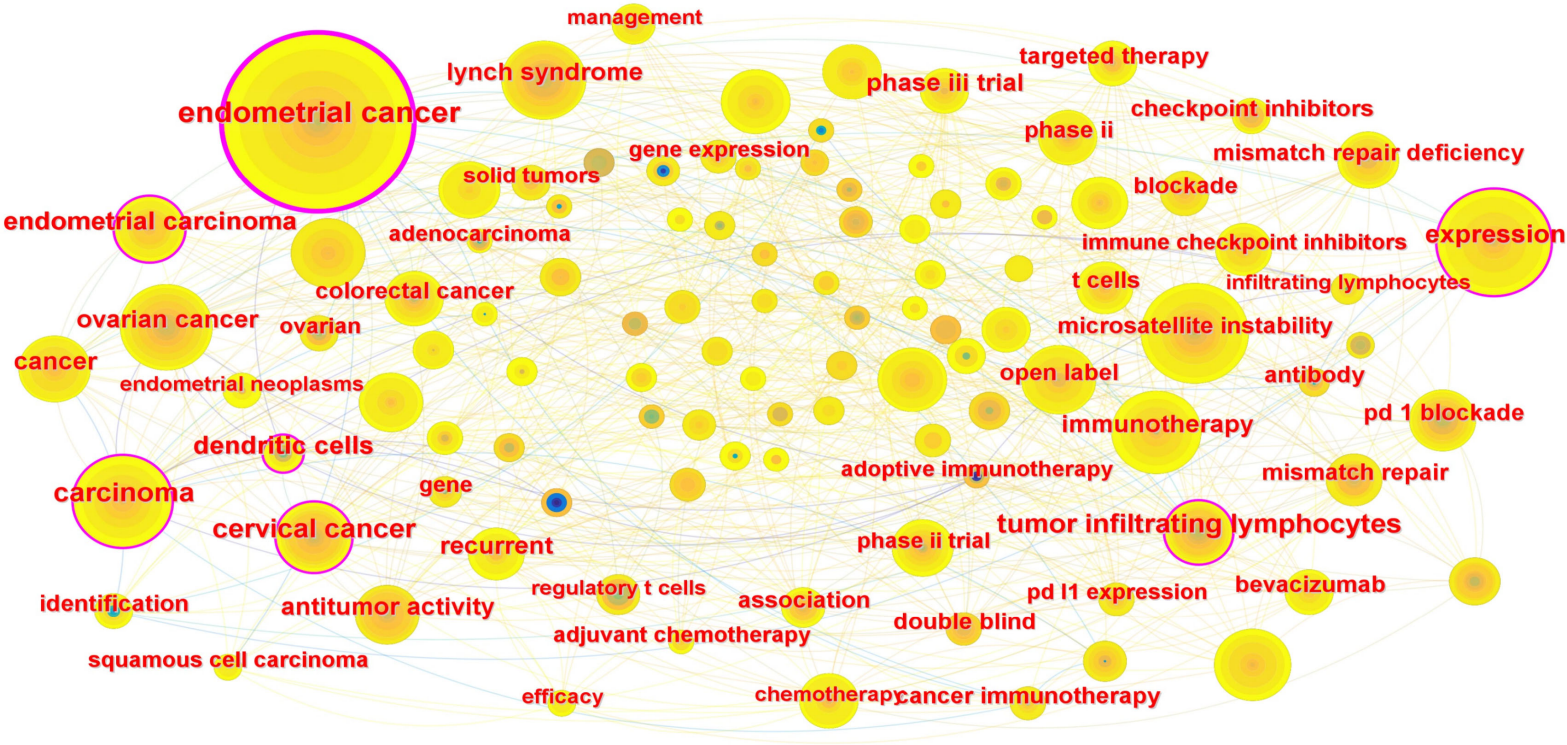
Keyword co-occurrence network of publications on immunotherapy for endometrial cancer. The network was generated by CiteSpace based on the co-occurrence analysis of keywords from 836 publications. Nodes represent keywords, with node size corresponding to their frequency of occurrence. The connections between nodes indicate co-occurrence relationships in the same article. The keywords were grouped into three major clusters: (1) *Endometrial cancer* — representing the disease background; (2) *Immunotherapy* — reflecting therapeutic approaches including immune checkpoint blockade; and (3) *Genetic factors* — such as microsatellite instability (MSI) and Lynch syndrome, indicating the genetic basis and biomarkers associated with immunotherapy responsiveness. This network highlights the major research focuses in the field.

Keyword co-occurrence analysis was performed using CiteSpace to characterize major research themes within the field. Three dominant clusters were identified: (1) endometrial cancer disease biology, (2) immunotherapy and immune checkpoint blockade, and (3) genetic and molecular determinants, including microsatellite instability and Lynch syndrome. As illustrated in **Figure 4**, these clusters reflect the convergence of clinical, immunologic, and genomic perspectives, indicating that research activity is increasingly oriented toward precision immunotherapy approaches.

#### 4.1.6 Timeline of keywords

**Figure 5:**
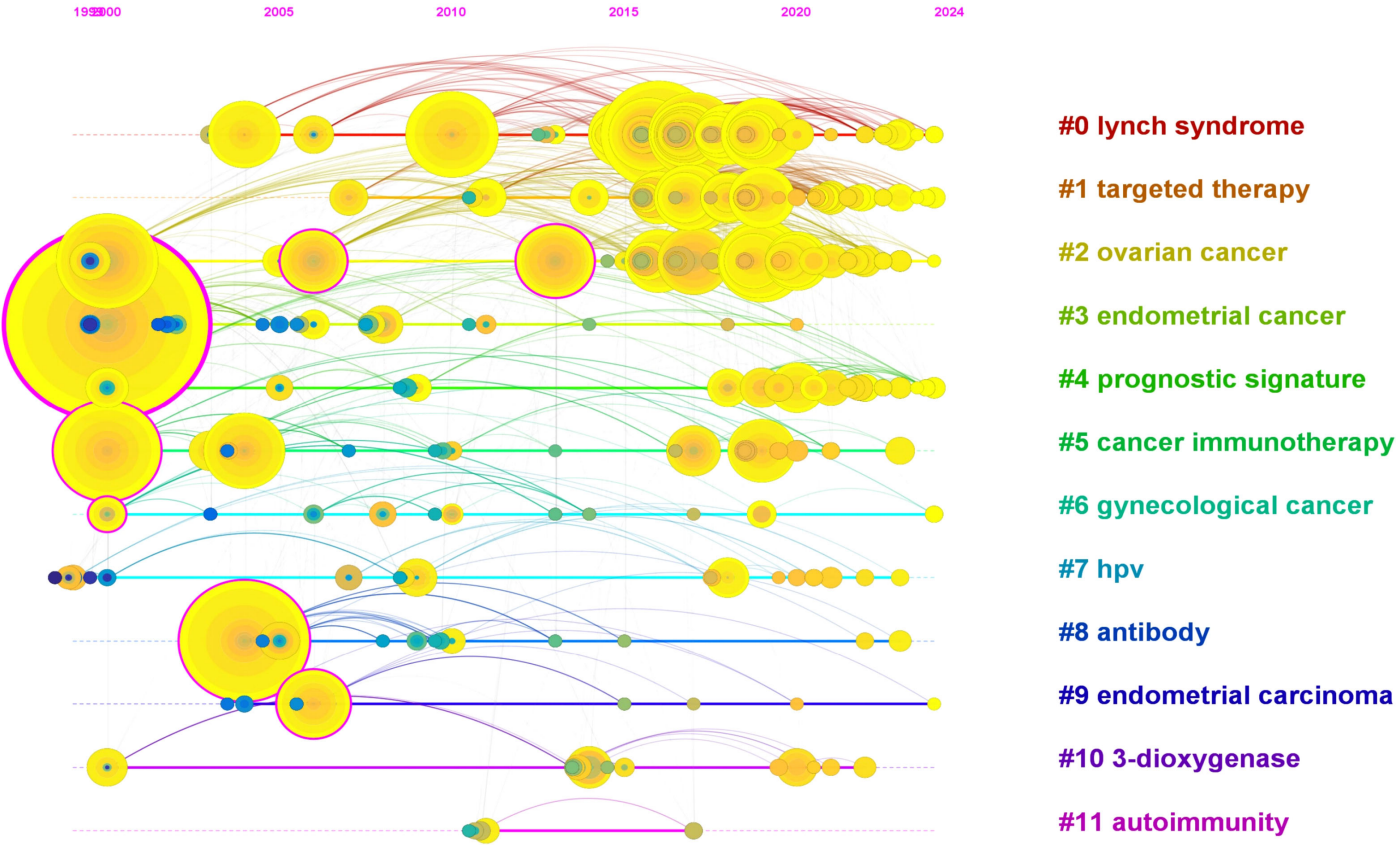
Timeline visualization of keyword clusters in the field of immunotherapy for endometrial cancer. The timeline view was generated by CiteSpace based on keyword clustering from 836 publications. Each horizontal line represents a distinct keyword cluster, labeled with the most representative term. The position of nodes on the timeline corresponds to the first appearance of the keyword in the literature, while the size of the nodes reflects their frequency of occurrence. In total, 11 major clusters were identified, including #0 Lynch syndrome, #1 Targeted therapy, #2 Ovarian cancer, #3 Endometrial cancer, #4 Prognostic signature, #5 Cancer immunotherapy, #6 Gynecological cancer, #7 HPV, #8 Antibody, #9 Endometrial carcinoma, #10 3-dioxygenase, and #11 Autoimmunity. Clusters with lower numbers indicate higher relevance or larger size. The timeline view reveals the temporal evolution and research hotspots in the field.

Timeline visualization of keyword clusters revealed how research priorities have evolved over time. Eleven major clusters were identified, including #0 lynch syndrome, #1 targeted therapy, #2 ovarian cancer, #3 endometrial cancer, #4 prognostic signature, #5 cancer immunotherapy, #6 gynecological cancer, #7 HPV, #8 antibody, #9 endometrial carcinoma, #10 3-dioxygenase, and #11 autoimmunity (**Figure 5**). Early research primarily focused on hereditary risk and molecular pathogenesis (e.g., Lynch syndrome), whereas more recent clusters emphasize immunotherapy, genomic biomarkers, and prognostic modeling. This temporal progression suggests a shift from descriptive disease characterization toward mechanistically informed therapeutic development.7. Keyword burst analysis.

**Figure 6:**
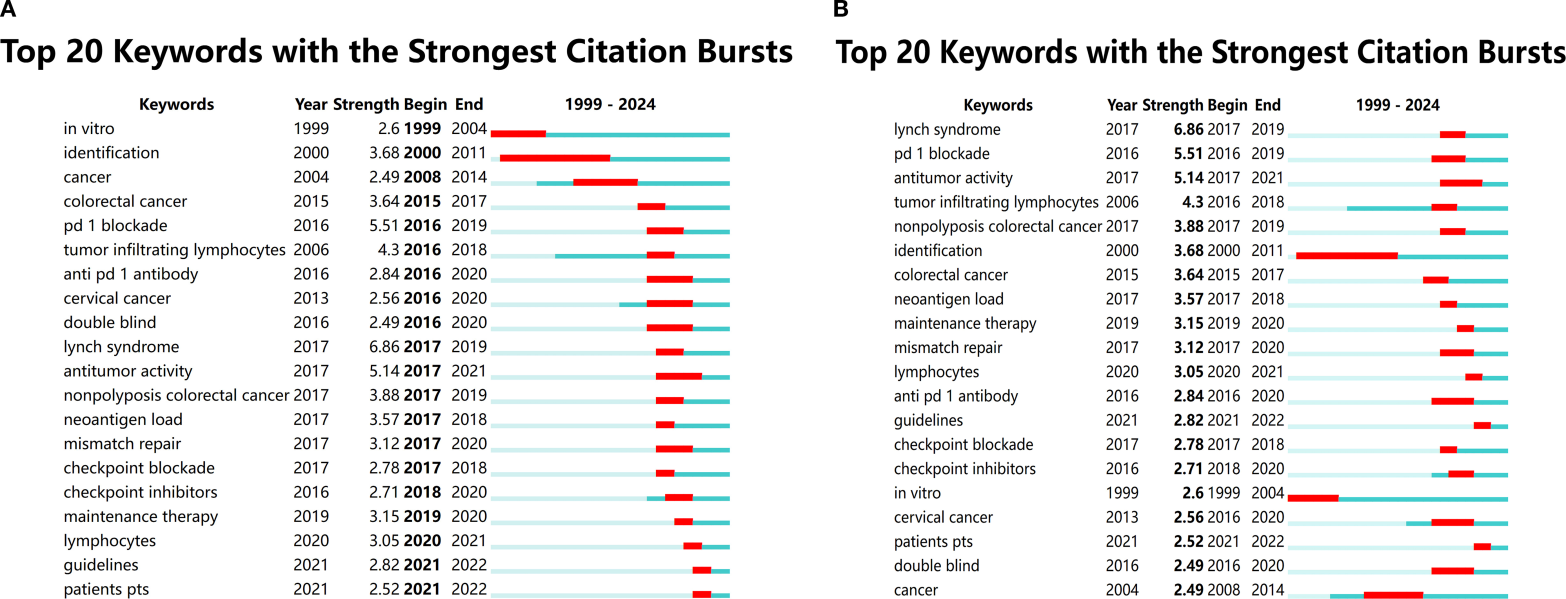
Keyword burst analysis based on Kleinberg’s algorithm. **(A)** Chronological distribution of the top 20 burst keywords identified in endometrial cancer immunotherapy research. Each colored segment represents the active period of a burst keyword over time. **(B)** Ranking of the top 20 burst keywords by burst intensity. The strongest burst was observed for “Lynch syndrome” (6.86), indicating its high impact in the field between 2017 and 2019.The burst keywords reflect two major research focuses: genetic predisposition to endometrial cancer (e.g., Lynch syndrome) and tumor immune microenvironment and immunotherapy strategies (e.g., PD-1 blockade, neoantigen load), highlighting evolving research trends in the field.

Keyword burst detection, based on Kleinberg’s algorithm (11), identified terms that experienced rapid surges in scholarly attention. Among the 99 burst keywords, the top 20 were selected and ranked according to duration and intensity (**Figures 6A, B**). The strongest burst occurred for “lynch syndrome” (strength = 6.86, active from 2017 to 2019), highlighting the pivotal role of genetic predisposition in defining risk stratification and immunotherapy responsiveness. Other notable bursts included “tumor infiltrating lymphocytes,” “neoantigen load,” and “PD-1 blockade,” reflecting growing interest in tumor microenvironment dynamics and molecular predictors of immunotherapy efficacy. These burst patterns underscore the field’s transition toward biomarker-driven immunotherapeutic strategies in endometrial cancer.

### 4.2 Clinical trials

#### 4.2.1 Clinical trial development

**Figure 7:**
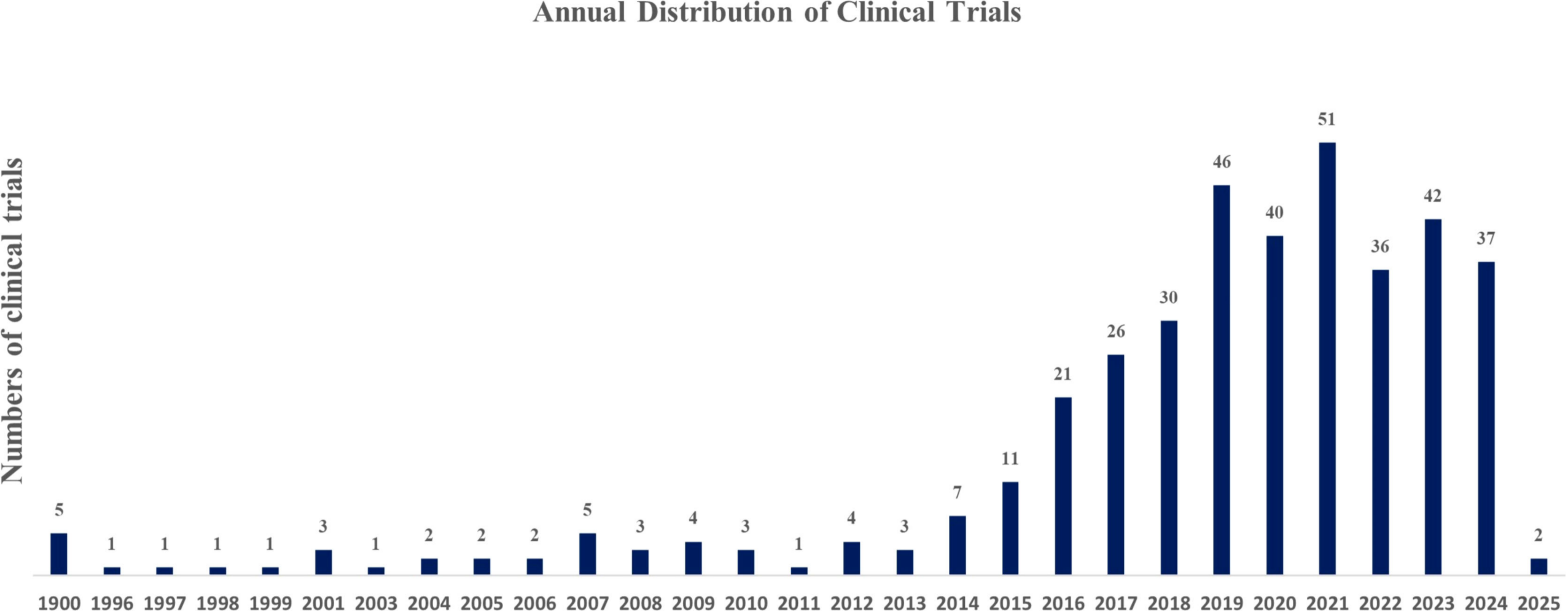
Annual trend of clinical trials on immunotherapy for endometrial cancer. The figure presents the yearly distribution of registered clinical trials involving immunotherapy for endometrial cancer from 1997 to 2024, based on data collected from ClinicalTrials.gov and ICTRP. Early-stage trials began in 1997, primarily exploring IL-2 class agents, GM-CSF therapies, and cancer vaccines targeting Ras protein. After a prolonged period of limited activity, the emergence of immune checkpoint inhibitors around 2015 marked a significant acceleration in clinical trial activity, peaking in 2021. The trend suggests growing interest and a promising future for novel immunotherapy strategies in endometrial cancer treatment.

Clinical trial activity related to immunotherapy for endometrial cancer began as early as 1997 with early cytokine-based approaches, including IL-2, GM-CSF, and Ras-targeted cancer vaccines. However, progress remained limited for nearly two decades, reflecting the absence of effective immune targets and the slow translation of immunobiology into therapeutic development. A marked inflection point occurred after 2015, coinciding with the clinical adoption of PD-1/PD-L1 inhibitors across multiple tumor types. From this period onward, the number of immunotherapy trials increased rapidly and reached a peak in 2021, indicating a substantial acceleration of research investment and drug development in this field (**Figure 7**). Linear regression confirmed a significant upward trend in annual trial registrations from 2005 to 2025 (β = 2.12 trials per year, p = 1.3 × 10^-4^, R² = 0.55), indicating a sustained expansion of clinical activity in this field.

**Figure 8:**
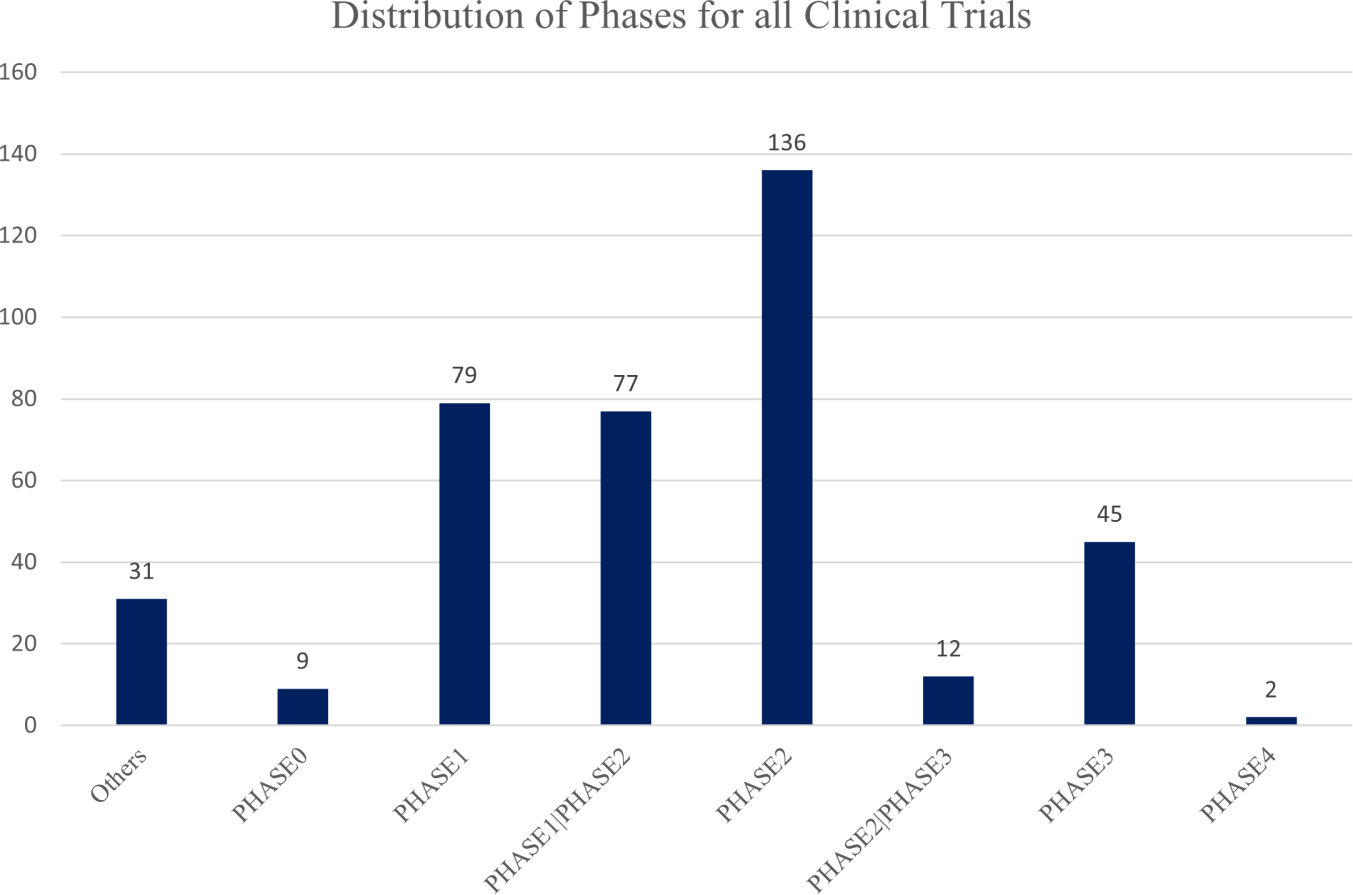
Phase distribution of clinical trials on immunotherapy for endometrial cancer. The figure illustrates the classification of 391 registered clinical trials on endometrial cancer immunotherapy by study phase. Most trials were concentrated in Phase I (n = 156) and Phase II (n = 213), reflecting early- and mid-stage clinical evaluations of novel therapies. Additionally, 45 trials reached Phase III, indicating advanced-stage studies aimed at assessing efficacy and potential clinical application in endometrial cancer treatment.

Across the 392 registered trials, most studies were early phase, including Phase I (n = 156) and Phase II (n = 213), while 45 Phase III trials were identified (**Figure 8**). This distribution reflects both the exploratory nature of immunotherapy in endometrial cancer and the ongoing transition toward late-phase evaluation of combination strategies with demonstrated early-phase efficacy.

#### 4.2.2 Immunotherapy strategies for endometrial cancer

**Figure 9:**
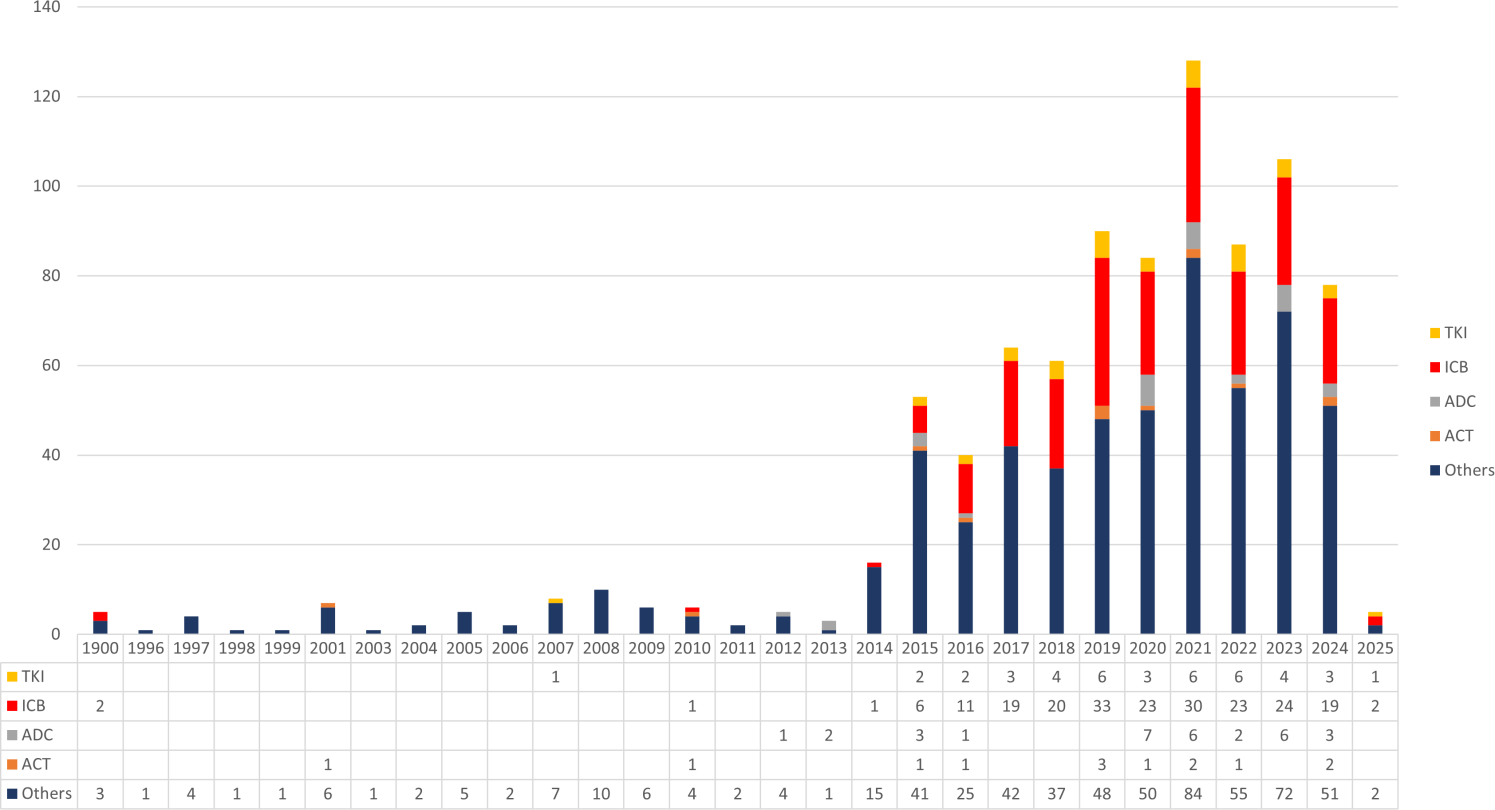
Classification and distribution of therapeutic strategies for endometrial cancer immunotherapy. The figure summarizes the main treatment modalities applied in endometrial cancer, including chemotherapy, endocrine therapy, antibody-drug conjugates (ADCs), anti-angiogenic therapy, and immunotherapy. Among these, immune checkpoint blockade (ICB) has emerged as the dominant immunotherapeutic approach in recent years. Additionally, the use of tyrosine kinase inhibitors (TKIs) and ADCs has shown a gradual increase. The figure also illustrates the classification of therapies based on targeted mechanisms and clinical application trends.

Despite established modalities such as surgery, radiotherapy, chemotherapy, and hormonal therapy, outcomes for advanced and recurrent endometrial cancer remain poor. Molecular characterization has revealed substantial heterogeneity within EC, with dMMR/MSI-H tumors displaying an inflamed tumor microenvironment, high neoantigen load, and strong susceptibility to PD-1 blockade. In contrast, pMMR/MSS tumors exhibit immune-excluded phenotypes and derive minimal benefit from PD-1 monotherapy. These biologic differences have directly shaped modern immunotherapy development and highlight the need for mechanistically informed therapeutic strategies (**Figure 9**).

Analysis of intervention strategies across the 392 registered clinical trials demonstrated marked heterogeneity in therapeutic focus, reflecting the divergent immunologic landscapes of EC. ICB–centered regimens accounted for the majority of studies, whereas targeted therapy, adoptive cell therapy, and vaccine-based approaches were explored primarily to address the limited responsiveness of pMMR/MSS tumors. The distribution of therapeutic modalities was highly non-uniform (χ² = 367.4, p = 2.3×10^-75^), indicating a strong global preference for ICB-based strategies. This skewed distribution suggests that combination immunotherapy is being actively pursued to overcome the biologic constraints of PD-1 monotherapy in the pMMR subgroup.

#### 4.2.3 Immunotherapeutic drugs

**Figure 10:**
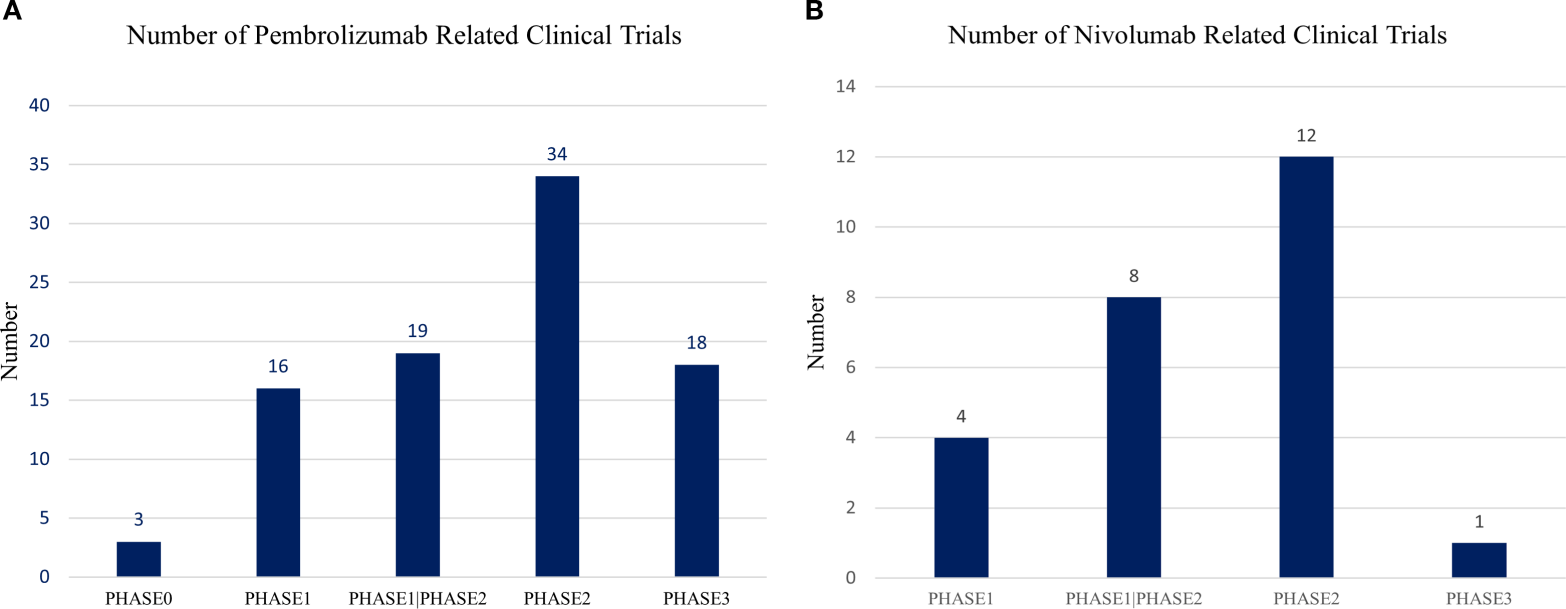
Phase distribution of clinical trials involving immune checkpoint inhibitors in endometrial cancer. **(A)** Clinical trials involving pembrolizumab. Pembrolizumab, as the first immune checkpoint inhibitor (ICI) applied to endometrial cancer, has been included in 92 registered clinical trials. Among these, 34 are Phase II and 18 are Phase III studies, reflecting its central role in immunotherapy for endometrial cancer. **(B)** Clinical trials involving nivolumab. A total of 25 clinical trials have investigated nivolumab, with 12 Phase II trials and 1 Phase III trial. Nivolumab is frequently administered in combination with other ICIs or chemotherapy, predominantly in Phase I or II studies. Three of these trials have reported clinical outcomes to date.

Pembrolizumab is the most extensively investigated immunotherapeutic agent in endometrial cancer, with 92 registered clinical trials, including 34 Phase II studies and 18 Phase III studies (**Figure 10A**). Its predominant use reflects the strong biological rationale for targeting PD-1 in dMMR/MSI-H tumors and the drug’s early regulatory success in multiple tumor types. Nivolumab has also been widely explored, with 25 trials identified—12 in Phase II and one in Phase III (**Figure 10B**). Most nivolumab studies evaluate combination strategies, including regimens incorporating other ICB or chemotherapy, consistent with efforts to enhance activity in pMMR/MSS tumors where monotherapy activity remains limited.

Research interest in bispecific antibodies has increased rapidly in recent years. Several emerging agents are now undergoing clinical evaluation, including the PD-L1 × 4-1BB bispecific DuoBody^®^-PD-L1x4-1BB. Early findings from a Phase II trial (CTIS2022-502453-33-00) combining this bispecific antibody with pembrolizumab have shown encouraging immunologic activity, suggesting that bispecific engagement of checkpoint and costimulatory pathways may offer a new therapeutic avenue for biologically resistant subtypes of endometrial cancer.

## 5 Discussion

By integrating a scientometric analysis of 836 publications with a comprehensive landscape review of 392 registered clinical trials, this study provides a multidimensional view of the evolution of immunotherapy in endometrial cancer. Rather than examining individual therapeutic modalities in isolation, our analysis reveals convergent patterns linking research output, clinical development, and regulatory progress. Notably, peaks in publication activity closely parallel key clinical milestones, including the approval of immune checkpoint inhibitors for MSI-H/dMMR disease and the emergence of combination strategies targeting immunologically “cold” tumors. These observations indicate that progress in the field has been driven not merely by incremental research accumulation, but by translational breakthroughs that reshaped therapeutic feasibility.

Building on these overarching trends, a central insight emerging from this integrated analysis is the persistent divergence in immunotherapy efficacy across molecular subtypes of endometrial cancer. While immune checkpoint blockade has demonstrated durable clinical benefit in MSI-H/dMMR tumors, therapeutic responses in the predominant pMMR/MSS population remain limited. This disparity is reflected consistently across clinical trial outcomes, publication patterns, and the expanding focus on combination regimens. Collectively, these findings highlight a critical shift in the field—from consolidating immune checkpoint inhibition in biologically favorable subgroups toward addressing the unmet need posed by molecularly “cold,” immunotherapy-resistant disease.

This study provides a comprehensive overview of the scientometric landscape and clinical trial development of immunotherapy in endometrial cancer. Research activity has expanded substantially over the past decade, with China and the United States emerging as the leading contributors to publication output. Among individual investigators, Lorusso D produced the highest number of publications, while Pignata S had the highest citation frequency and Le DT exhibited the highest co-citation count. Notably, nine of the fifteen most co-cited references were clinical trials, including six evaluating pembrolizumab, two Phase I studies of the bispecific antibody dostarlimab, and one Phase II trial of lenvatinib plus pembrolizumab. These patterns underscore the pivotal role of clinical evidence in shaping the conceptual framework of immunotherapy for endometrial cancer.

Keyword-based analyses—including co-occurrence mapping, timeline visualization, and burst detection—demonstrated a clear evolution from early interest in hereditary risk (e.g., Lynch syndrome) toward contemporary focus on tumor immunogenicity, neoantigen load, tumor-infiltrating lymphocytes, PD-1 blockade, and combination immunotherapy. These findings highlight how genomic characterization and immune-microenvironment insights have guided therapeutic innovation in this field.

**Figure 11:**
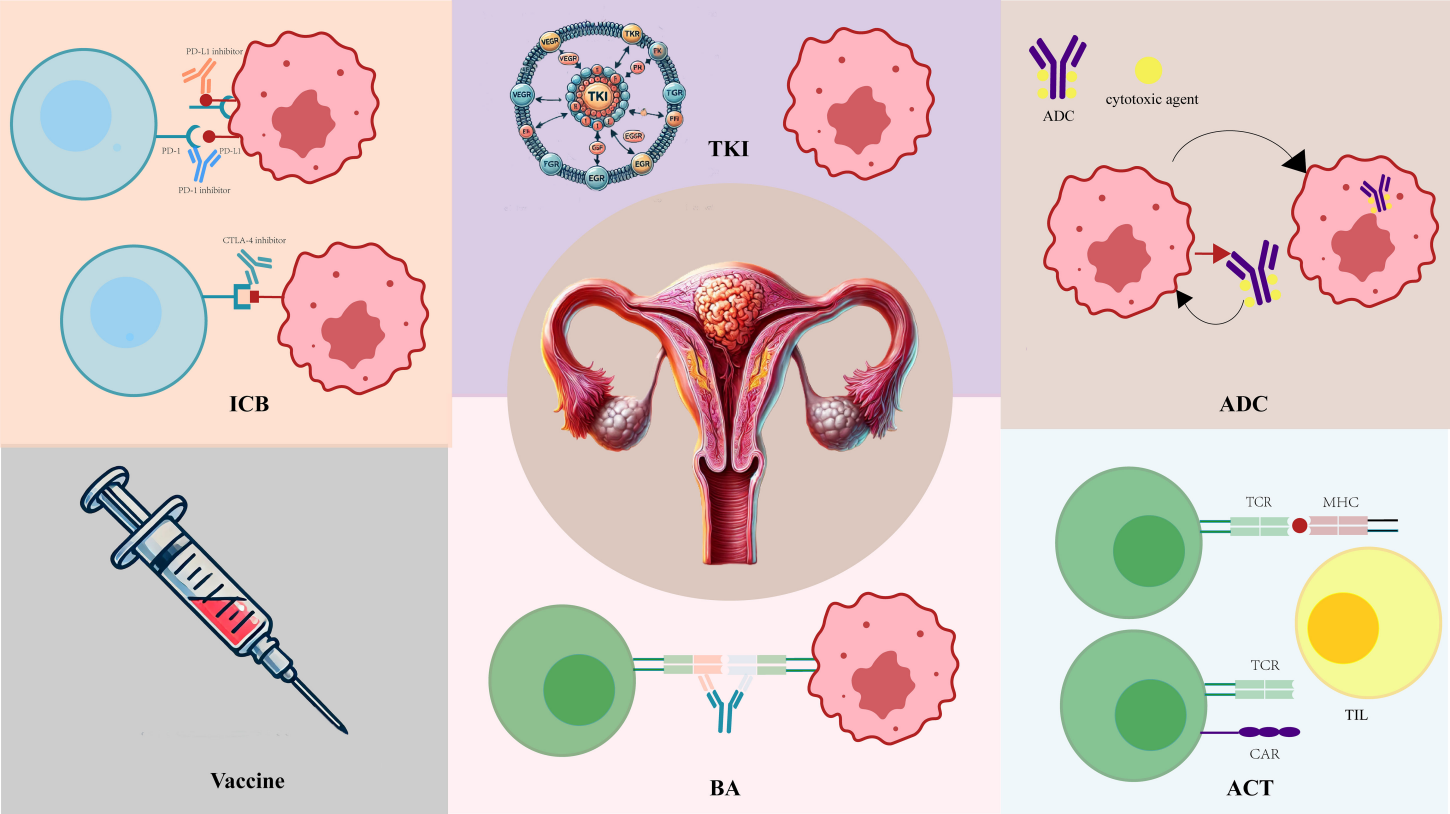
Schematic representation of various therapeutic strategies targeting endometrial cancer. The central image illustrates the anatomy of the endometrium as the primary site of disease. ICB (Immune Checkpoint Blockade): Depicts the inhibition of immune checkpoints like PD-1/PD-L1 and CTLA-4, which are proteins that regulate immune responses. Inhibiting these checkpoints reactivates T-cells, enhancing their ability to target and kill tumor cells. Vaccine: Represents vaccine-based strategies aimed at generating immune responses against tumor-associated antigens. This approach stimulates the body’s immune system to recognize and attack cancer cells. BA (Bispecific Antibodies): Illustrates the mechanism of bispecific antibodies, which are engineered to simultaneously bind to T-cells and tumor cells. This dual binding facilitates targeted immune responses, bringing T-cells into close proximity to tumor cells to promote cell killing. ACT (Adoptive Cell Transfer): Shows the process of adoptive cell therapy, where T-cells, including tumor-infiltrating lymphocytes, are isolated, expanded, and reintroduced to the patient to target and destroy cancer cells. ADC (Antibody-Drug Conjugates): Demonstrates antibody-drug conjugates, where antibodies are linked to cytotoxic agents. Upon binding to tumor-specific antigens, ADCs deliver cytotoxic agents directly to the cancer cells, leading to their destruction. TKI (Tyrosine Kinase Inhibitors): Illustrates targeted therapy using tyrosine kinase inhibitors, which block signaling pathways essential for cancer cell proliferation and survival, resulting in cell death. Each of these therapeutic approaches represents a distinct mechanism for targeting uterine cancers, either through immune modulation, direct cytotoxicity, or inhibition of essential signaling pathways.

Multiple immunotherapeutic strategies are now being explored, including immune-checkpoint inhibitors, monoclonal antibodies, adoptive cell therapy, and combination regimens integrating ICBs with targeted agents such as PARP inhibitors or PI3Kδ inhibitors. The breadth of these strategies reflects the biological diversity of endometrial cancer, particularly the differential responsiveness of dMMR/MSI-H versus pMMR/MSS tumors. **Figure 11** summarizes the major immunotherapy modalities currently under investigation.

Overall, the rapidly expanding clinical trial portfolio and the emergence of novel modalities, including bispecific antibodies and rational ICB-based combinations, suggest a promising trajectory for the development of precision immunotherapy in endometrial cancer.

### 5.1 ICB

ICB has become the foundational immunotherapy strategy in endometrial cancer. PD-1 is expressed on activated T cells, while PD-L1 is broadly expressed on tumor cells and immune cells. Engagement of PD-1 with PD-L1 suppresses T-cell activation, allowing tumor immune escape. PD-1 inhibitors (e.g., pembrolizumab, nivolumab) block this interaction to restore T-cell function, whereas PD-L1 inhibitors (e.g., atezolizumab, durvalumab) directly target tumor-expressed PD-L1 to prevent immune suppression (12). ICB monotherapy has shown meaningful activity, particularly in MSI-H/dMMR tumors. Pembrolizumab achieved an ORR of 48% (95% CI 37–60) with durable responses (13). while dostarlimab demonstrated an ORR of 42.3% in post-platinum dMMR EC (14). Nivolumab monotherapy produced an ORR of 36% with manageable toxicity (15).

In China, several PD-1 inhibitors, including serplulimab, tislelizumab, envafolimab, and pucotenlimab, have shown preliminary clinical activity. In particular, serplulimab achieved an ORR of 38.2% and a 12-month OS rate of 81.2% (16). Tislelizumab achieved an ORR of 19% in MSI-H/dMMR tumors (17). Studies of envafolimab and pucotenlimab have also included small endometrial cancer cohorts (n=6 and n=7, respectively), providing early signals of clinical activity (18, 19).

These findings establish ICB monotherapy as the backbone for combination strategies. The following subsections summarize key ICB-based combinations currently under evaluation, including chemotherapy, radiotherapy, TKIs, and bispecific antibodies.

#### 5.1.1 ICB and chemotherapy

Mounting preclinical evidence indicates that cytotoxic chemotherapy can potentiate ICB by increasing tumor antigen release, upregulating PD-L1 expression, and disrupting stromal barriers to facilitate T-cell infiltration. These immunomodulatory effects provide a strong mechanistic rationale for combining chemotherapy with ICB in endometrial cancer (12).

Clinical evidence has confirmed this synergy. In a randomized Phase III trial, pembrolizumab plus paclitaxel and carboplatin achieved a 12-month progression-free survival(PFS) rate of 74% in dMMR endometrial cancer, markedly superior to 38% in the placebo group (HR, 0.30; 95% CI, 0.19–0.48; P<0.001). Among patients with pMMR tumors, median PFS improved from 8.7 months with placebo to 13.1 months with pembrolizumab (HR, 0.54; 95% CI, 0.41–0.71; P<0.001), with a safety profile consistent with pembrolizumab and standard chemotherapy (20).

Similarly, in a global Phase III study of dostarlimab plus carboplatin and paclitaxel, the dMMR–MSI-H cohort showed a 24-month PFS of 61.4% versus 15.7% with placebo (HR, 0.28; 95% CI, 0.16–0.50; P<0.001). In the overall population, 24-month PFS improved from 18.1% to 36.1% (HR, 0.64; 95% CI, 0.51–0.80), and OS from 56.0% to 71.3% (HR, 0.64; 95% CI, 0.46–0.87) (21). Another Phase III trial demonstrated significant PFS benefits with durvalumab alone (HR, 0.71; 95% CI, 0.57–0.89; P = 0.003) or durvalumab plus olaparib (HR, 0.55; 95% CI, 0.43–0.69; P<0.0001), with consistent benefit across dMMR, pMMR, and PD-L1–positive subgroups (22).

Among the 58 trials in our dataset evaluating ICB–chemotherapy combinations, several early-phase studies have explored the addition of PD-1 inhibitors to cytotoxic or anti-angiogenic regimens. The Phase II trial NCT02549209 is assessing pembrolizumab combined with carboplatin and paclitaxel in advanced or recurrent endometrial adenocarcinoma (23), whereas NCT02853318 (24) is evaluating pembrolizumab in combination with bevacizumab and low-dose cyclophosphamide in recurrent gynecologic cancers. Although full peer-reviewed efficacy data are not yet available for these studies, their design reflects the growing rationale for integrating PD-1 blockade with chemotherapy or anti-angiogenic therapy to enhance antitumor activity in recurrent or advanced EC.

#### 5.1.2 ICB and radiotherapy

Radiotherapy (RT) generates immunogenic cell death, enhances neoantigen presentation, and promotes dendritic cell activation, thereby creating an immune-permissive environment that may augment the efficacy of ICB (25). The potential for synergistic activity provides the rationale for combining RT with PD-1/PD-L1 blockade in endometrial cancer.

Our dataset includes eight ongoing trials evaluating ICB–RT combinations. NCT04214067 (26) is a Phase III trial comparing pembrolizumab plus adjuvant RT versus RT alone in newly diagnosed high-risk early-stage endometrial cancer. ChiCTR2300078191 (27) is a Phase II trial examining cadonilimab combined with RT versus RT alone. As these trials are ongoing, efficacy data are not yet available. Although RT–ICB combinations exhibit strong mechanistic and early clinical promise, optimal sequencing, dosing, and toxicity management remain key areas for future investigation.

#### 5.1.3 ICB and TKIs

Tyrosine kinase inhibitors (TKIs), particularly multi-targeted VEGFR inhibitors, modulate both angiogenesis and immune suppression by reducing regulatory T cells and myeloid-derived suppressor cells while normalizing tumor vasculature. These effects enhance cytotoxic T-cell infiltration and create a biologically favorable context for ICB. This mechanistic rationale underlies the extensive clinical exploration of TKI–ICB combinations in endometrial cancer. The Phase II study (NCT02501096) demonstrated that lenvatinib plus pembrolizumab produced meaningful responses in advanced or recurrent endometrial cancer, with a safety profile consistent with each agent individually, aside from higher rates of hypothyroidism (28).

In the pivotal Phase III NCT03517449 trial, lenvatinib plus pembrolizumab significantly improved outcomes in predominantly pMMR/MSS patients: median PFS was 7.2 months versus 3.8 months with chemotherapy, ORR was 31.9% versus 14.7%, and median OS improved to 17.4 months versus 12.0 months, confirming superior clinical benefit over standard chemotherapy (29).

NCT02912572 further explored avelumab alone or combined with talazoparib (PARP inhibitor) or axitinib (TKI) in recurrent or persistent endometrial cancer, reporting an SDR of 57.1% and median stable-disease duration of 3.8 months (30).

Among the 37 trials in our dataset evaluating TKI–ICB combinations, lenvatinib and axitinib were the most frequently used TKIs, reflecting their established anti-angiogenic and immunomodulatory profiles.

#### 5.1.4 Bispecific antibody

Bispecific antibodies (BsAbs) offer the ability to simultaneously modulate multiple immune pathways, thereby overcoming the limited activity observed with single-target checkpoint inhibitors. By engaging dual immune mechanisms and enhancing selective activation within the tumor microenvironment, BsAbs can improve anti-tumor responses while minimizing off-target toxicity (31). Although single-agent PD-1 or CTLA-4 inhibitors have provided meaningful benefits across several malignancies, their efficacy in biomarker-unselected endometrial cancer remains modest (32), underscoring the need for next-generation immunotherapies.

Cadonilimab, a PD-1/CTLA-4 bispecific antibody, has emerged as a leading agent in this class. In our dataset, 12 trials evaluated BsAbs, most frequently cadonilimab either as monotherapy or in combination with other ICBs. Growing clinical experience highlights its potential to broaden benefit beyond traditional MSI-H/dMMR populations. GEN1046 is another bispecific agent under investigation, designed to block PD-L1 while conditionally activating 4-1BB, thereby integrating checkpoint inhibition with costimulatory signaling. In a Phase II trial (CTIS2022-502453-33-00) combining GEN1046 with pembrolizumab in advanced endometrial cancer, the disease control rate reached 65.6% (40/61), including responses in patients previously resistant to PD-L1 inhibitors, suggesting activity in ICB-refractory disease.

Ivonescimab (AK112), a PD-1/VEGF-A bispecific antibody, also demonstrated early efficacy signals. Among 47 evaluable patients, the confirmed ORR was 25.5% (12/47), with a disease control rate of 63.8% (30/47). Notably, responses were observed in both dMMR and pMMR endometrial cancer cohorts, supporting its potential to address resistance mechanisms beyond mismatch repair deficiency (33).

Collectively, the expanding portfolio of BsAbs—including cadonilimab, GEN1046, and ivonescimab—illustrates the rapid development of dual-targeting immunotherapies and their promise in overcoming current limitations of single-agent ICB in endometrial cancer.

### 5.2 ADC and immunotherapy

Antibody–drug conjugates (ADCs) represent one of the fastest-advancing classes of targeted therapeutics in oncology. An ADC consists of a monoclonal antibody linked to a cytotoxic payload through a chemical linker, enabling selective delivery of potent anti-tumor agents to cells expressing the target antigen and reducing systemic toxicity (34). After antigen binding, ADCs undergo receptor-mediated internalization and lysosomal degradation, releasing the active payload to induce cell death (35). However, their therapeutic efficacy may be limited by several resistance mechanisms—including insufficient antigen expression, impaired ADC internalization, lysosomal dysfunction, and alterations in drug efflux pathways—which remain critical barriers to broader clinical application (36).

Several ADCs targeting HER2 have shown early evidence of anti-tumor activity in endometrial cancer. Trastuzumab deruxtecan (T-DXd) demonstrated a 55% response rate and a median PFS of 6.2 months (95% CI, 4.0–8.8) in HER2-positive uterine serous carcinoma (37). SYD985 (trastuzumab duocarmazine), another HER2-directed ADC, achieved partial responses in 39% (5 of 13) of patients with metastatic endometrial cancer in a dose-expansion cohort, highlighting its potential for HER2-expressing EC (38).

Experience from other gynecologic malignancies further supports the promise of ADCs: in previously treated recurrent or metastatic cervical cancer, tisotumab vedotin achieved an objective response rate (ORR) of 24% (95% CI, 16–33), including 7% complete and 17% partial responses, providing durable and clinically meaningful activity (39).

Beyond HER2, novel ADCs targeting tumor-associated antigens such as AXL are being evaluated. Enapotamab vedotin, an AXL-directed ADC, has shown immune-stimulatory activity and tumor regression in humanized models of melanoma and lung cancer, providing a rationale for evaluating AXL-targeted ADCs in endometrial cancer, where clinical data remain scarce (40).Ongoing studies (NCT02552121, NCT02988817) will help clarify their relevance in EC (41, 42).

Combination regimens involving ADCs and ICB represent a particularly promising direction. ADC-mediated tumor cell lysis can increase antigen release, enhance dendritic-cell activation, and potentially synergize with PD-1/PD-L1 inhibitors. Several early-phase trials—including NCT04278144 (43), evaluating nivolumab combined with an ADC, and NCT04486352 (44), assessing ADCs with atezolizumab—are exploring this strategy.

In total, 31 ADC-related clinical trials were identified in our dataset. Although most are in early phases and lack published efficacy results, the growing diversity of targets and increasing integration with immunotherapy highlight a rapidly expanding—yet still under-validated—therapeutic frontier in endometrial cancer.

### 5.3 Vaccine therapy

Therapeutic cancer vaccines aim to enhance antitumor immunity by improving antigen presentation and restoring effective T-cell responses. These strategies rely on efficient delivery of immunogenic antigens to dendritic cells (DCs), activation of DC-mediated priming, and sustained expansion of CD4^+^ and CD8^+^ T cells (45). Although early DC-based vaccines demonstrated proof of concept, recent advances in neoantigen discovery and immune profiling have renewed interest in vaccine approaches, particularly when combined with immune checkpoint blockade.

Endometrial cancers with MSI-H represent an especially compelling setting for vaccine development, given their characteristic accumulation of frameshift mutations and enriched neoantigen landscapes. Neoantigens represent non-self tumor proteins with high immunogenicity that can be recognized by both CD4+ and CD8+ T cells, providing a mechanistic basis for personalized and shared antigen-targeted cancer vaccines (45).

Clinical translation in endometrial cancer remains limited but active. A Phase I/II trial (NCT05269381) (46) is currently evaluating a neoantigen peptide vaccine in combination with GM-CSF and immune checkpoint inhibitors. Across registered trials, eight studies involving vaccine strategies were identified, although no efficacy results have yet been reported.

Overall, vaccine-based immunotherapy remains an emerging modality in endometrial cancer. While the high neoantigen load of MSI-H/dMMR tumors provides a strong biological rationale, significant challenges persist, including reliable antigen prediction, efficient intratumoral T-cell recruitment, and integration with existing immunotherapeutic regimens. Nonetheless, vaccine approaches represent a promising component of future combination strategies aimed at overcoming resistance to checkpoint blockade and enhancing durable immune control.

### 5.4 Adoptive cell therapy

Adoptive cell therapy (ACT) encompasses multiple personalized immunotherapeutic platforms—including tumor-infiltrating lymphocytes (TILs), chimeric antigen receptor T (CAR-T) cells, and TCR-engineered T cells—designed to enhance antitumor immunity through the direct infusion of expanded or genetically modified effector lymphocytes (47, 48).

Advances in ex vivo T-cell expansion, gene-transfer technologies, and lymphodepletion regimens have significantly broadened the applicability of ACT in solid tumors, although translation to endometrial cancer (EC) remains at an early stage. Across our dataset, 12 ACT-related trials were identified, reflecting growing interest but underscoring persistent challenges such as identifying optimal tumor-specific targets, ensuring long-term persistence of transferred cells, and overcoming the profoundly immunosuppressive tumor microenvironment characteristic of EC.

#### 5.4.1 TILs

TIL-based immunotherapy has demonstrated clinical benefit in tumors such as melanoma and cervical cancer, inspiring exploratory evaluation in EC. All three included EC-related TIL trials were Phase I or II studies, focusing on IL-2–supported expansion strategies or combinations with PD-1 blockade (e.g., pembrolizumab), though no results have yet been reported.

#### 5.4.2 CAR-T

CAR-T cells, which recognize surface antigens independently of MHC presentation, offer a theoretical advantage for tumors with low MHC expression and resistance to TCR-based strategies (49).

An early Phase I study evaluating anti-ALPP CAR-T cells (TC-A101) demonstrated preliminary antitumor activity, including tumor regression and one partial response, without severe treatment-related toxicity (50).

However, CAR-T translation in EC remains limited due to heterogeneous antigen expression and concerns regarding on-target/off-tumor toxicity, particularly in aggressive type II EC subtypes (51).

#### 5.4.3 TCR-T

T-cell receptor–engineered T cells (TCR-T) can recognize intracellular tumor antigens presented via pMHC and are more sensitive to low-copy epitopes than CAR-T cells, offering a conceptual advantage for solid tumors (52).

In a Phase I trial (NCT04044859) (53), ADP-A2M4CD8 T cells targeting MAGE-A4 demonstrated promising antitumor activity with confirmed objective responses observed in heavily pretreated solid tumors, along with durable disease stabilization in select patients. An ongoing Phase I/II study (NCT05194735) (54) is further evaluating TCR-T in combination with IL-2 support to enhance T-cell activity. Integrated Interpretation of Therapeutic Strategies.

Beyond direct immune checkpoint inhibition, increasing evidence suggests that tumor microenvironmental factors—including stromal composition, angiogenic signaling, and immune cell exclusion—play a critical role in shaping immunotherapy responsiveness in endometrial cancer. This broader immunologic context provides a unifying mechanistic rationale for the expanding exploration of combination strategies observed across recent clinical trials. Such observations are consistent with emerging evidence that tumor–immune interactions, stromal architecture, and immune cell exclusion critically shape responsiveness to immunotherapy across solid tumors (55–57).

Viewed collectively, the therapeutic strategies discussed in this review reflect an ongoing effort to overcome primary resistance to immunotherapy through rational combinations and novel immune-engaging platforms. Chemotherapy, radiotherapy, targeted agents, antibody–drug conjugates, and adoptive cell therapies are increasingly investigated not as standalone solutions, but as complementary approaches designed to enhance tumor immunogenicity, remodel the tumor microenvironment, or amplify antitumor immune responses. The concentration of recent trials in these areas mirrors both mechanistic advances and the limitations revealed by earlier single-agent studies. Beyond pharmacological combinations, recent representative studies have explored diverse biology-informed strategies that can reshape tumor behavior and immune responsiveness (e.g., modulation of tumor–stroma/immune interactions and immune-conditioning approaches), highlighting the expanding conceptual landscape of cancer immunotherapy (58–61).

Importantly, this integrated scientometric and clinical trial mapping highlights the value of combining quantitative research trend analysis with outcome-oriented clinical evidence. Such an approach allows not only the identification of dominant therapeutic paradigms, but also the recognition of gaps where clinical activity has outpaced biological understanding or vice versa. As immunotherapy in endometrial cancer continues to evolve, future progress will likely depend on molecularly informed trial design, biomarker-driven patient stratification, and combination strategies tailored to specific resistance mechanisms. By contextualizing clinical development within broader research and regulatory trajectories, this study provides a framework for understanding past progress and guiding future innovation in the field. This bidirectional interaction between mechanistic research and clinical trial design reflects a broader translational paradigm in contemporary cancer immunotherapy, in which evolving biological insights increasingly inform patient selection, therapeutic sequencing, and endpoint definition in clinical studies. More broadly, the translation of accumulating clinical trial evidence into practice-oriented guidelines represents a critical step in maximizing the real-world impact of emerging immunotherapy strategies. Guideline-oriented syntheses and re-conceptualization efforts provide an important framework for bridging clinical evidence with standardized care pathways in rapidly evolving oncologic fields (62).

## 6 Conclusion and future perspectives

This review integrates, a scientometric mapping of 836 publications with a clinical trial landscape analysis of 392 registered studies in endometrial cancer immunotherapy, providing a multidimensional overview of research output, therapeutic evolution, and translational progress. Together, these datasets illustrate an accelerating global shift toward immune-based strategies—particularly PD-1/PD-L1 blockade—driven by expanding clinical evidence, regulatory momentum, and deepening mechanistic insight into tumor–immune interactions.

Our meta-analytic synthesis further quantifies the magnitude of therapeutic disparity between molecular subgroups. dMMR/MSI-H tumors exhibit a pooled ORR of approximately 52.2% (95% CI, 41.5–62.7), whereas pMMR/MSS tumors demonstrate markedly lower responses (15.1%, 95% CI, 10.6–21.1). These findings reinforce the biological foundations of response heterogeneity in endometrial cancer and highlight the urgent need for new strategies that can effectively target the molecularly “cold” pMMR/MSS majority, consistent with established mechanistic and clinical evidence in this field (4, 5).

Scientometric patterns parallel the clinical trajectory of the field. Peaks in publication output emerged around landmark approvals—such as pembrolizumab for MSI-H/dMMR disease—and major trial readouts. Geographic trends similarly mirrored sponsorship of clinical investigations, underscoring a reciprocal relationship between scientific visibility and therapeutic development. At the same time, our analysis exposes persistent imbalances, with immune checkpoint blockade dominating the trial portfolio, while vaccines, bispecific antibodies, and adoptive cell therapies remain underrepresented despite their strong mechanistic rationale. Recent clinical advancements further support these trends. Multiple large randomized studies have shown that integrating PD-1 blockade into first-line or recurrent treatment yields substantial benefit in MSI-H/dMMR tumors and provides incremental gains in selected pMMR/MSS populations when combined with chemotherapy or anti-angiogenic agents. Although these studies were published beyond the upper limit of our bibliometric window, they reinforce the trajectory identified by our analyses and highlight the increasingly central role of immunotherapy in EC management.

Looking ahead, the successful clinical translation of immunotherapy in EC will depend on refinements in biomarker-based patient selection, rational combination strategies, and deeper mechanistic understanding of immune escape. Broadening therapeutic benefit beyond MSI-H/dMMR tumors will require identification of predictive features such as neoantigen load, immune-gene signatures, tumor microenvironmental determinants, and multi-omics-defined immune phenotypes. Similarly, thoughtful combination approaches—integrating ICB with TKIs, anti-angiogenic agents, radiotherapy, ADCs, bispecific antibodies, or adoptive cellular therapies—offer promising avenues to overcome resistance in the refractory pMMR/MSS subgroup. Advances in single-cell immunology, spatial profiling, and systems-level integration will be essential for refining these therapeutic strategies and guiding personalized-treatment design.

Several limitations should be acknowledged. Scientometric analyses depend on indexing accuracy and may underrepresent non-English or unpublished literature. Clinical trial registries contain variable levels of reporting detail, and survival endpoints (PFS, OS) were insufficiently available for quantitative synthesis across modalities. Nonetheless, the integrated framework presented here offers a comprehensive, evidence-based appraisal of the rapidly evolving immunotherapy landscape in endometrial cancer.

Collectively, these findings signal a therapeutic transition—from monotherapy checkpoint inhibition toward biomarker-driven, multimodal, and mechanistically informed immunotherapy. As research investment grows and novel technologies mature, personalized immune-based strategies are poised to deliver meaningful and durable benefit for patients with endometrial cancer. By combining scientometric insight with clinical evidence, this review provides a foundation for understanding emerging priorities and guiding the next phase of translational and clinical innovation in the field.

## 7 Limitations

This study has several limitations that should be acknowledged. First, the scientometric component relied exclusively on the Web of Science Core Collection, which may not fully capture relevant publications indexed in other databases such as Scopus or PubMed, potentially leading to selection bias. Second, although 392 endometrial cancer immunotherapy trials were identified, a considerable proportion lacked published outcomes or had incomplete reporting in trial registries, limiting the ability to comprehensively synthesize efficacy or safety data across modalities. Third, substantial heterogeneity in study design, patient populations, eligibility criteria, and clinical endpoints across trials complicates cross-study comparisons and may introduce interpretative bias. Fourth, while our meta-analysis provides quantitative estimates of ORR differences between dMMR/MSI-H and pMMR/MSS tumors, the analysis was restricted by the limited availability of subgroup-level response data and the absence of consistent survival endpoints (PFS, OS), precluding broader meta-analytic integration. Finally, as immunotherapy in endometrial cancer is a rapidly evolving field, emerging therapeutic modalities and newly published clinical outcomes may not have been captured within the time frame of our data collection.

Such limitations are inherent to landscape-based and registry-driven analyses, particularly when integrating bibliometric mapping with trial registry data across heterogeneous study designs. Similar constraints related to data availability, reporting completeness, and interpretative heterogeneity have been widely recognized in large-scale translational and biomedical synthesis studies, where they may influence the generalizability of integrated evidence (56, 63).
